# Supplementary material for: Humor Styles, Bullying Victimization and Psychological School Adjustment: Mediation, Moderation and Person-Oriented Analyses
Source: Int J Environ Res Public Health. 2022 Sep 10;19(18):11415. doi: 10.3390/ijerph191811415 (PMC9517355; doi:10.3390/ijerph191811415)
Supplement: Supplementary file 1 [file ijerph-19-11415-s001.zip › ijerph-1879595-supplementary.pdf]

International Journal of
Environmental Research and Public Health

MDPI

Supplementary Materials

Humor Styles, Bullying Victimization and Psychological School Adjustment: Mediation, Moderation and Person-Oriented Analyses

Christoph Burger

Supplementary Table S1. Parameter estimates of the model with victimization mediating the association between humor styles and psychological school adjustment.

						95% CI			
**Predictors**	**Outcome**	**Estimate**	***SE***	***z***	***p***	**Lower**	**Upper**	***Std* (all)**	***Std* (nox)**
School adjustment	Victimization	**−0.383*****	0.061	−6.305	< 0.001	−0.502	−0.264	−0.415	−0.415
School adjustment	Aggressive humor	0.069	0.076	0.906	0.365	−0.080	0.217	0.058	0.058
School adjustment	Affiliative humor	0.099	0.072	1.374	0.170	−0.042	0.241	0.087	0.087
School adjustment	Self−enhancing humor	**0.289*****	0.076	3.823	< 0.001	0.141	0.438	0.242	0.242
School adjustment	Self−defeating humor	**−0.338*****	0.073	−4.641	< 0.001	−0.481	−0.196	−0.316	−0.316
Victimization	Aggressive humor	−0.158	0.096	−1.641	0.101	−0.346	0.031	−0.122	−0.122
Victimization	Affiliative humor	**−0.190***	0.092	−2.069	0.039	−0.369	−0.010	−0.153	−0.153
Victimization	Self−enhancing humor	−0.032	0.097	−0.326	0.744	−0.222	0.158	−0.024	−0.024
Victimization	Self−defeating humor	**0.299*****	0.091	3.293	< 0.001	0.121	0.478	0.258	0.258
Aggressive humor	Age	0.027	0.039	0.672	0.502	−0.051	0.104	0.050	0.024
Aggressive humor	Class conflict frequency	0.123	0.094	1.309	0.191	−0.061	0.308	0.098	0.110
Aggressive humor	Gender	**0.579****	0.201	2.873	0.004	0.184	0.974	0.215	0.515
Affiliative humor	Age	−0.002	0.042	−0.055	0.956	−0.084	0.080	−0.004	−0.002
Affiliative humor	Class conflict frequency	0.174‡	0.100	1.738	0.082	−0.022	0.370	0.132	0.148
Affiliative humor	Gender	0.248	0.214	1.158	0.247	−0.171	0.667	0.088	0.211
Self−enhancing humor	Age	**0.081***	0.040	2.054	0.040	0.004	0.159	0.156	0.073
Self−enhancing humor	Class conflict frequency	−0.128	0.095	−1.349	0.177	−0.314	0.058	−0.102	−0.114
Self−enhancing humor	Gender	0.102	0.203	0.503	0.615	−0.295	0.499	0.038	0.091
Self−defeating humor	Age	−0.035	0.044	−0.805	0.421	−0.120	0.050	−0.060	−0.028
Self−defeating humor	Class conflict frequency	**0.361*****	0.104	3.468	< 0.001	0.157	0.565	0.258	0.289
Self−defeating humor	Gender	−0.068	0.222	−0.305	0.760	−0.504	0.368	−0.023	−0.054
Victimization	Age	−0.037	0.047	−0.785	0.432	−0.129	0.055	−0.054	−0.025
Victimization	Class conflict frequency	**0.601*****	0.116	5.182	< 0.001	0.374	0.829	0.370	0.414
Victimization	Gender	−0.004	0.242	−0.016	0.988	−0.478	0.471	−0.001	−0.003
School adjustment	Age	−0.004	0.037	−0.105	0.917	−0.076	0.068	−0.006	−0.003
School adjustment	Class conflict frequency	−0.150	0.098	−1.538	0.124	−0.341	0.041	−0.100	−0.112
School adjustment	Gender	0.053	0.189	0.279	0.780	−0.317	0.423	0.016	0.039

Note. Calculated with JASP[51]. Delta method standard errors; full information maximum likelihood estimator. *Std* = standardized estimates; significant vales ($p \leq 0.05$) are displayed in bold.
‡ $p \leq 0.10$, * $p \leq 0.05$, ** $p \leq 0.01$, *** $p \leq 0.001$

Supplementary Table S2. Aggressive humor style: Conditional effect of the focal predictor victimization on school adjustment.

Aggressive humor (moderator)	Conditional Effects	Heteroscedasticity-consistent *SE*	*t* value	*p* value	Lower limit 95% *CI*	Upper limit 95% *CI*
1.000 (−2.037)	**−0.593*** **	0.118	−5.047	< 0.001	−0.825	−0.361
1.270 (−1.767)	**−0.554*** **	0.105	−5.292	< 0.001	−0.761	−0.347
1.540 (−1.497)	**−0.515*** **	0.093	−5.548	< 0.001	−0.698	−0.332
1.810 (−1.227)	**−0.476*** **	0.082	−5.785	< 0.001	−0.638	−0.313
1.911 (−1.126)[1]	**−0.461*** **	0.079	−5.857	< 0.001	−0.617	−0.306
2.080 (−0.957)	**−0.437*** **	0.074	−5.935	< 0.001	−0.582	−0.291
2.350 (−0.687)	**−0.397*** **	0.068	−5.885	< 0.001	−0.531	−0.264
2.620 (−0.417)	**−0.358*** **	0.065	−5.522	< 0.001	−0.486	−0.230
2.890 (−0.147)	**−0.319*** **	0.066	−4.834	< 0.001	−0.450	−0.189
3.037 (0.000)[2]	**−0.298*** **	0.068	−4.367	< 0.001	−0.432	−0.163
3.160 (0.123)	**−0.280*** **	0.071	−3.956	< 0.001	−0.420	−0.140
3.430 (0.393)	**−0.241** **	0.078	−3.069	0.003	−0.396	−0.086
3.700 (0.663)	**−0.202* **	0.088	−2.282	0.024	−0.376	−0.027
3.820 (0.783)	**−0.184* **	0.093	−1.976	0.050	−0.368	0.000
3.970 (0.933)	−0.162	0.100	−1.628	0.106	−0.360	0.035
4.163 (1.126)[3]	−0.134	0.109	−1.238	0.218	−0.349	0.080
4.240 (1.203)	−0.123	0.112	−1.098	0.274	−0.345	0.098
4.510 (1.473)	−0.084	0.126	−0.670	0.504	−0.332	0.164
4.780 (1.743)	−0.045	0.139	−0.323	0.747	−0.320	0.230
5.050 (2.013)	−0.006	0.153	−0.038	0.970	−0.309	0.297
5.320 (2.283)	0.033	0.168	0.199	0.843	−0.298	0.365
5.590 (2.553)	0.073	0.183	0.397	0.692	−0.288	0.433
5.860 (2.823)	0.112	0.197	0.566	0.572	−0.278	0.502
6.130 (3.093)	0.151	0.212	0.710	0.479	−0.269	0.571
6.400 (3.363)	0.190	0.228	0.835	0.405	−0.259	0.640

Note. Numbers in brackets represent mean-centered values. The PROCESS Macro[52] (model 1) was used with a heteroscedasticity consistent standard error (Huber-White) to obtain the Johnson-Neyman output. The value 3.820 (0.783) of the moderator variable aggressive humor defines the upper boundary of the Johnson-Neyman significance region (77.25% of values below, 22.75% above). Significant vales ($p \leq 0.05$) are displayed in bold.

[1] *M* – 1 *SD*; [2] *M*, [3] *M* + 1 *SD*

* $p \leq 0.05$, ** $p \leq 0.01$, *** $p \leq 0.001$

Supplementary Table S3. Self-defeating humor style: Conditional effect of the focal predictor victimization on school adjustment.

Self-defeating humor (moderator)	Conditional Effects	Heteroscedasticity-consistent *SE*	*t* value	*p* value	Lower limit 95% *CI*	Upper limit 95% *CI*
1.000 (−2.228)	0.029	0.131	0.219	0.827	−0.230	0.288
1.300 (−1.928)	−0.015	0.121	−0.123	0.902	−0.253	0.223
1.600 (−1.628)	−0.058	0.110	−0.529	0.597	−0.277	0.160
1.900 (−1.328)	−0.102	0.101	−1.013	0.313	−0.301	0.097
1.979 (−1.249)[1]	−0.113	0.098	−1.154	0.250	−0.308	0.081
2.200 (−1.028)	−0.146	0.092	−1.588	0.114	−0.327	0.036
2.377 (−0.851)	**−0.171***	0.087	−1.976	0.050	−0.343	0.000
2.500 (−0.728)	**−0.189***	0.083	−2.268	0.025	−0.354	−0.024
2.800 (−0.428)	**−0.233****	0.076	−3.052	0.003	−0.383	−0.082
3.100 (−0.128)	**−0.276*****	0.071	−3.920	< 0.001	−0.416	−0.137
3.228 (0.000)[2]	**−0.295*****	0.069	−4.301	< 0.001	−0.430	−0.159
3.400 (0.173)	**−0.320*****	0.067	−4.810	< 0.001	−0.451	−0.189
3.700 (0.473)	**−0.363*****	0.065	−5.625	< 0.001	−0.491	−0.236
4.000 (0.773)	**−0.407*****	0.065	−6.260	< 0.001	−0.535	−0.279
4.300 (1.073)	**−0.451*****	0.068	−6.659	< 0.001	−0.584	−0.317
4.477 (1.249)[3]	**−0.476*****	0.070	−6.786	< 0.001	−0.615	−0.338
4.600 (1.373)	**−0.494*****	0.072	−6.834	< 0.001	−0.637	−0.351
4.900 (1.673)	**−0.538*****	0.079	−6.841	< 0.001	−0.693	−0.382
5.200 (1.973)	**−0.581*****	0.086	−6.745	< 0.001	−0.752	−0.411
5.500 (2.273)	**−0.625*****	0.095	−6.596	< 0.001	−0.812	−0.438
5.800 (2.573)	**−0.668*****	0.104	−6.425	< 0.001	−0.874	−0.463
6.100 (2.873)	**−0.712*****	0.114	−6.252	< 0.001	−0.937	−0.487
6.400 (3.173)	**−0.756*****	0.124	−6.086	< 0.001	−1.001	−0.510
6.700 (3.473)	**−0.799*****	0.135	−5.930	< 0.001	−1.065	−0.533
7.000 (3.773)	**−0.843*****	0.146	−5.787	< 0.001	−1.130	−0.555

Note. Numbers in brackets represent mean-centered values. The PROCESS Macro[52] (model 1) was used with a heteroscedasticity consistent standard error (Huber-White) to obtain the Johnson-Neyman output. The value 2.377 (−0.851) of the moderator variable self-defeating humor defines the lower boundary of the Johnson-Neyman significance region (23.35% of values below, 76.65% above). Significant vales ($p \leq 0.05$) are displayed in bold.

[1] *M* − 1 *SD*; [2] *M*, [3] *M* + 1 *SD*

* $p \leq 0.05$, ** $p \leq 0.01$, *** $p \leq 0.001$

Supplementary Table S4. Comparison of prevalence and demographic information of the bullying-related groups.

**Statistics**	**Noninvolved**	**Pure victims**	**Bully-victims**	**Comparison results**	
**Chi² Goodness of Fit: Frequency comparison**				$X^2(2)$	
Sample size (%)	125 (74.0%)	23 (13.6%)	21 (12.4%)	**125.586*** **	
Number of females (%)	96 (76.8%)	17 (73.9%)	16 (76.2%)	0.100	
**ANOVA: Mean comparison**				$F(2,166)$	η_p^2
Mean age *(SD)*	22.72 (2.16)	22.04 (2.08)	22.71 (2.05)	0.993	0.012
Mean frequency of conflicts in class (*SD*)	2.74 (0.78)	3.39 (1.12)	3.43 (1.03)	**9.807*** **	0.106

Note. Significant values ($p \leq 0.05$) are displayed in bold.
*** $p \leq 0.001$

Supplementary Table S5. Comparison of fit indices in latent profile models with different numbers of classes.

Fit indices	2-class latent profile	3-class latent profile	4-class latent profile	5-class latent profile
AIC	2131.43	2107.28	2107.79	2103.54
BIC	2172.35	2163.93	2180.18	2191.67
entropy	0.77	0.74	0.74	0.76
$prob_{min}$	0.84	0.86	0.75	0.66
$prob_{max}$	0.96	0.90	0.92	0.92
n_{min}	0.23	0.22	0.13	0.07
n_{max}	0.77	0.50	0.49	0.42
BLRT p-value	0.01	0.01	0.17	0.10

Note. AIC = "Akaike information criterion"; BIC = "Bayesian information criterion"; BLRT = "Bootstrap likelihood ratio test"; $prob_{min}$ = "minimum of the average latent class probabilities for most likely class membership"; $prob_{max}$ = "maximum of the average latent class probabilities for most likely class membership"; n_{min} = "proportion of the sample assigned to the smallest class"; n_{max} = "proportion of the sample assigned to the largest class". Analyses were calculated with Jamovi[54], using the snowRMM module[55] which is based on the tidyLPA R package[56].

Supplementary Table S6. Tukey post hoc tests on different humor styles for the three humor-related groups.

**Post-hoc comparison of aggressive humor**		**Mean Difference**	***SE***	***t***	**Cohen's *d***	p_{tukey}
overall high (class 1)	adaptive high (class 2)	0.894	0.211	**4.233*****	0.839	< 0.001
	adaptive low (class 3)	0.544	0.236	2.301‡	0.512	0.058
adaptive high (class 2)	adaptive low (class 3)	–0.351	0.193	–1.817	–0.327	0.167
**Post-hoc comparison of affiliative humor**						
overall high (class 1)	adaptive high (class 2)	0.44	0.134	**3.275****	0.672	0.004
	adaptive low (class 3)	2.42	0.15	**16.098*****	3.468	< 0.001
adaptive high (class 2)	adaptive low (class 3)	1.98	0.123	**16.119*****	2.948	< 0.001
**Post-hoc comparison of self-enhancing humor**						
overall high (class 1)	adaptive high (class 2)	0.497	0.203	**2.452***	0.473	0.040
	adaptive low (class 3)	1.313	0.227	**5.795*****	1.303	< 0.001
adaptive high (class 2)	adaptive low (class 3)	0.816	0.185	**4.41*****	0.803	< 0.001
**Post-hoc comparison of self-defeating humor**						
overall high (class 1)	adaptive high (class 2)	2.378	0.155	**15.319*****	3.091	< 0.001
	adaptive low (class 3)	2.113	0.174	**12.168*****	2.43	< 0.001
adaptive high (class 2)	adaptive low (class 3)	–0.266	0.142	–1.873	–0.337	0.150

Note. Significant values ($p \leq 0.05$) are displayed in bold.
‡ $p \leq 0.10$, * $p \leq 0.05$, ** $p \leq 0.01$, *** $p \leq 0.001$
